# Rethinking Thin-Layer Chromatography for Screening Technetium-99m Radiolabeled Polymer Nanoparticles

**DOI:** 10.1021/acsptsci.4c00383

**Published:** 2024-08-30

**Authors:** Kathrin Schorr, Xinyu Chen, Takanori Sasaki, Anahi Paula Arias-Loza, Johannes Lang, Takahiro Higuchi, Achim Goepferich

**Affiliations:** † Department of Pharmaceutical Technology, University of Regensburg, Regensburg, Bavaria 93053, Germany; ‡ Nuclear Medicine, Faculty of Medicine, University of Augsburg, Augsburg, Bavaria 86156, Germany; § Department of Nuclear Medicine and Comprehensive Heart Failure Center, University Hospital Würzburg, Würzburg, Bavaria 97080, Germany; ∥ Faculty of Medicine, Dentistry and Pharmaceutical Sciences, Okayama University, Okayama 700-0082, Japan

**Keywords:** polymer nanoparticles, direct ^99m^Tc-labeling, single-photon emission computed tomography, radio-thin layer chromatography, radiocolloids

## Abstract

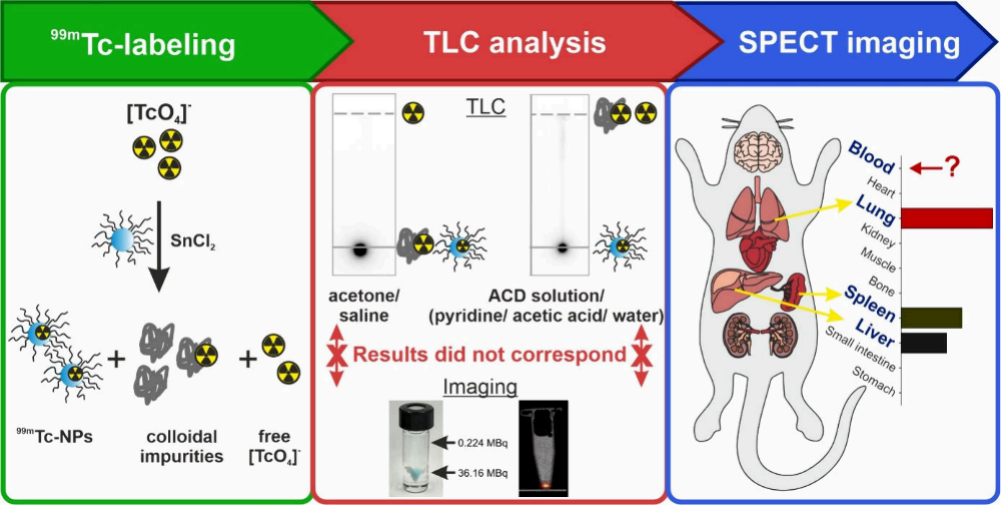

Thin-layer chromatography (TLC) is commonly employed to screen technetium-99m labeled polymer nanoparticle batches for unreduced pertechnetate and radio-colloidal impurities. Although this method is widely accepted, our findings applying radiolabeled PLGA/PLA–PEG nanoparticles underscore its lack of transferability between different settings and its limitations as a standalone quality control tool. While TLC profiles may appear similar for purified and radiocolloid containing nanoparticle formulations, their in vivo behavior can vary significantly, as demonstrated by discrepancies between TLC results and single-photon emission computed tomography (SPECT) and biodistribution data. This highlights the urgent need for a case-by-case evaluation of TLC methods for each specific nanoparticle type. Our study revealed that polymeric nanoparticles cannot be considered analytically uniform entities in the context of TLC analysis, emphasizing the complex interplay between nanoparticle composition, radiolabeling conditions, and subsequent biological behavior.

Nanoparticles (NPs) have emerged as a promising platform technology for drug delivery^1,2^ and diagnostics,^3^ but their unique properties pose challenges for traditional characterization methods like liquid chromatography–mass spectrometry. To overcome these limitations, radioactive labeling is often employed for in vivo studies, enabling real-time tracking with high sensitivity.^4,5^ Technetium-99m (^99m^Tc) is a popular radionuclide due to its suitable half-life, ideal photon energy for single-photon emission computed tomography (SPECT) imaging, and availability from ^99^Mo/^99m^Tc generators.^4^ For certain polymeric NPs, direct incorporation of radionuclides into the NP structure is feasible without additional chelating groups.^6−8^ However, this requires reducing pertechnetate (^99m^TcO_4_^–^) to a lower oxidation state.^9^ While tin chloride (SnCl_2_) is commonly used as a reducing agent^7,8,10−12^ due to its mild, nanoparticle-friendly reaction conditions,^6,9^ the radiolabeling process must be carefully optimized for each NP type. This optimization is crucial as various radiochemical impurities can occur during the labeling process, including unreduced ^99m^TcO_4_^–^ and different colloidal species such as ^99m^Tc(IV)-oxide-colloids and ^99m^Tc(IV)-Sn-colloids.^9,13^ Moreover, the formation of tin oxide and hydroxide colloids is suspected to cause attachment of the radiolabeled polymeric NPs and can further complicate the system.^14^ These colloidal impurities, collectively referred to as hydrolyzed-reduced technetium-99m (HR-^99m^Tc), can significantly impact radiolabeling efficiency and biodistribution data if undetected.^4^

To address this challenge, a comprehensive analysis of impurities in ^99m^Tc-NP batches is essential. Radio-thin layer chromatography (TLC) is a widely adopted method for assessing the radiochemical purity of radiopharmaceuticals due to its simplicity and speed.^15^ However, the application of TLC analysis to NPs is not standard practice in other fields.^16^ To distinguish ^99m^Tc-NPs from ^99m^TcO_4_^–^ and HR-^99m^Tc, two sets of developing systems for TLC analysis are commonly employed.^12,13,17,23^ An organic solvent (e.g., acetone or methyl ethyl ketone) and saline are used to identify ^99m^TcO_4_^–^. Subsequently, acidic developing solvents are utilized to separate ^99m^Tc-NPs from HR-^99m^Tc. Popular choices for these acidic solvents include anticoagulant citrate dextrose (ACD) solution (pH 5)^17^ and a pyridine, acetic acid, and water mixture (Py/AA/H_2_O).^11,23^ While ACD solution has been reported to develop HR-^99m^Tc, the Py/AA/H_2_O mixture is employed to develop ^99m^Tc-NPs (Figure 1). By comparing the results from both TLC plates, researchers can estimate the relative proportions of different species presented in the reaction mixture.^17^

**Figure 1:**
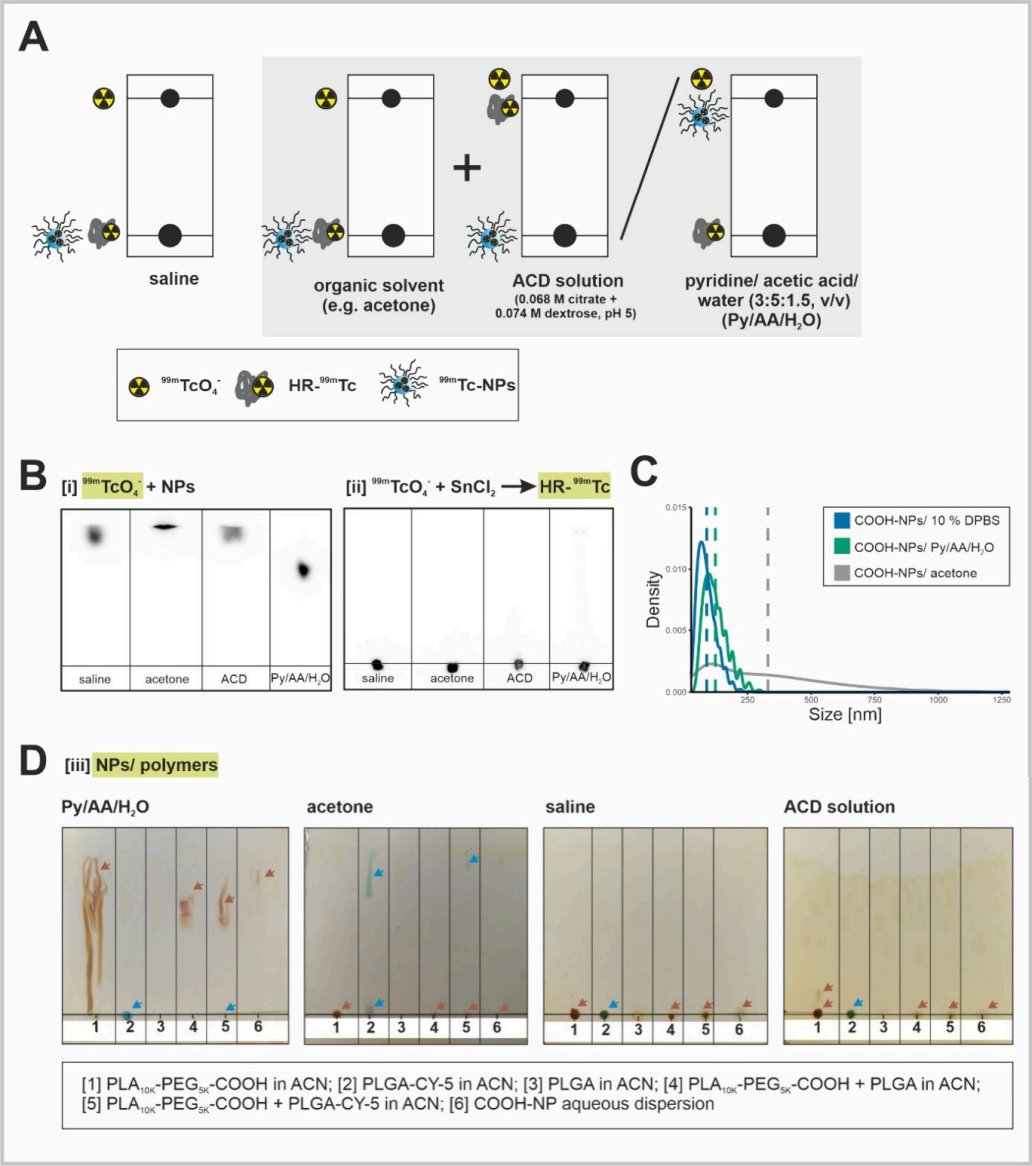
**Thin-layer chromatographic methods and control experiments.** (A) Illustration of the thin-layer chromatographic methods described in the literature^11,12,17,23^ for the analytical separation of ^99m^TcO_4_^–^, ^99m^Tc-NPs, and HR-^99m^Tc. (B) TLCs of NPs + ^99m^TcO_4_^–^ in the absence of a reducing agent and TLCs of ^99m^TcO_4_^–^ + SnCl_2_ (solubilized in HCl) in the absence of NPs. (C) Density plots of NP size distribution. The NPs were diluted with 10% DPBS, Py/AA/H_2_O, and acetone. Mean values are highlighted as vertical dashed lines corresponding to the color of the density curves. (D) TLCs of polymers and NPs developed in various developing solvents, followed by staining with iodine vapor. Blue arrows indicate spots visible before staining, while brown arrows represent spots revealed after iodine staining. PLGA [3] serves as a negative control for staining with iodine vapor.

The current study aims to examine and scrutinize the general applicability and transferability of the TLC analytical method outlined above. We selected polymeric poly(d,l-lactide-*co*-glycolide) (PLGA)/poly(ethylene glycol)-*b*-poly(d,l-lactide) (PLA–PEG) core–shell NPs for radiolabeling using ^99m^Tc and SnCl_2_, because they serve as a valuable model due to their composition of polymers that are widely used for NP production.^18,19^

## Results and Discussion

When establishing a ^99m^Tc radiolabeling protocol for cyanine-5-amine (CY-5)-tagged core–shell NPs using SnCl_2_ as a reducing agent, it was discovered that the formation of HR-^99m^Tc (Figure S1) was not reflected in the TLC analysis results (Figure S2), as per the adopted method from the literature (Figure 1A). Based on these observations, control experiments were conducted systematically to investigate the transferability of the TLC analysis. The developing of ^99m^TcO_4_^–^ ([i]), HR-^99m^Tc ([ii]), as well as NPs and their constituent polymers ([iii]) on the TLC plates using the solvents described in the literature was analyzed independently. Initially, NPs and ^99m^TcO_4_^–^ were investigated in the absence of SnCl_2_, confirming that unreacted ^99m^TcO_4_^–^ was developed with all of the solvents described (Figure 1B). As HR-^99m^Tc is mainly formed in the absence of a suitable ligand or in sufficient quantities of the ligand,^9^ the development behavior of a reaction mixture of ^99m^TcO_4_^–^ and SnCl_2_ without addition of NPs was analyzed (Figure 1B). The result showed that HR-^99m^Tc remained at the application point in all solvents, contradicting the previous reports,^17,20^ in which it was suggested that HR-^99m^Tc could be solubilized and developed with ACD solution due to complexation with the citrate it contains. Further orthogonal experiments using SnCl_2_ solubilized in citric acid instead of HCl confirmed the formation of a ^99m^Tc-citrate complex that could be developed with saline, ACD, and Py/AA/H_2_O mixture but not with acetone (Figure S3). A homogeneous distribution of radioactivity in the reaction vessel was observed, as recorded by a SPECT phantom (Figure S3). These findings emphasize that the formation of the ^99m^Tc-citrate complex is favored over the formation of HR-^99m^Tc and ^99m^Tc-NPs. Nevertheless, it cannot be assumed that such a complex forms quantitatively from HR-^99m^Tc and develops with citrate-containing ACD solution.

In addition, the development behavior of the NPs under consideration was analyzed. These NPs consist of a lipophilic PLGA core (Figure S4) and a relatively hydrophilic PLA–PEG copolymer shell (Figure S5), where the core and shell are attached by the lipophilic nature of PLGA and PLA in the aqueous nanoparticle dispersion (Figures S6 and S7). When applied to the TLC plates, polymeric NPs can no longer be considered as a singular analytical unit but rather as the sum of the polymers of which they are composed. The presumed disintegration of the nanoparticle structure was proven by dynamic light scattering (DLS) measurements of NPs diluted with organic solvents. The mean nanoparticle size shifted from 89.1 ± 36.2 nm (in 10% Dulbecco’s phosphate-buffered saline (DPBS)) to 123.3 ± 45.8 and 329.6 ± 242.3 nm when diluted with Py/AA/H_2_O mixture and acetone, respectively (Figure 1C, Figure S8). Therefore, in addition to NPs, polymer mixtures and individual polymers were also applied to the TLC plates to investigate their retention behavior. The fluorescent CY-5-tag facilitated the visual identification of labeled PLGA, while the PLA–PEG block-*co*-polymer was visualized on the TLC plates through iodine staining. The results indicate that purely aqueous solvents (saline and ACD solution) cannot develop the polymers or the NPs (Rf = 0), while mobile phases consisting of organic solvents lead to a partial development of the polymers (Rf = 0.2–0.8, Figure 1D, Figure S8). PLA–PEG was not developed in acetone (Rf = 0) but in a Py/AA/H_2_O mixture (Rf = 0.6–0.8). In contrast, PLGA was not developed in a Py/AA/H_2_O mixture (Rf = 0) but partially in acetone (Rf = 0.6–0.9). One reason for the partial development in acetone (Figure S8) could be the migration and separation of PLGA due to different molecular weights (Resomer RG 502, *M*_w_ 7000–17000).^21^ This was supported by the recorded HPLC chromatograms (Figure S4). Although the phase transition between the stationary and mobile phases is a driving force for the separation of the analytes during TLC development, these results cannot be explained simply by the solubility of the polymers in the mobile phases. An estimation of the solubility of both polymers in the solvents using the “Hansen parameters” showed a comparatively good solubility in the organic solvents used in the mobile phases (Figure S9). This shows that the retention in multicomponent systems depends not only on the solubility in the mobile phase but also on several other factors, such as association effects and the properties of the stationary phase.^22^ These results highlight the complexity of predicting the TLC behavior of polymeric systems.

**Figure 2:**
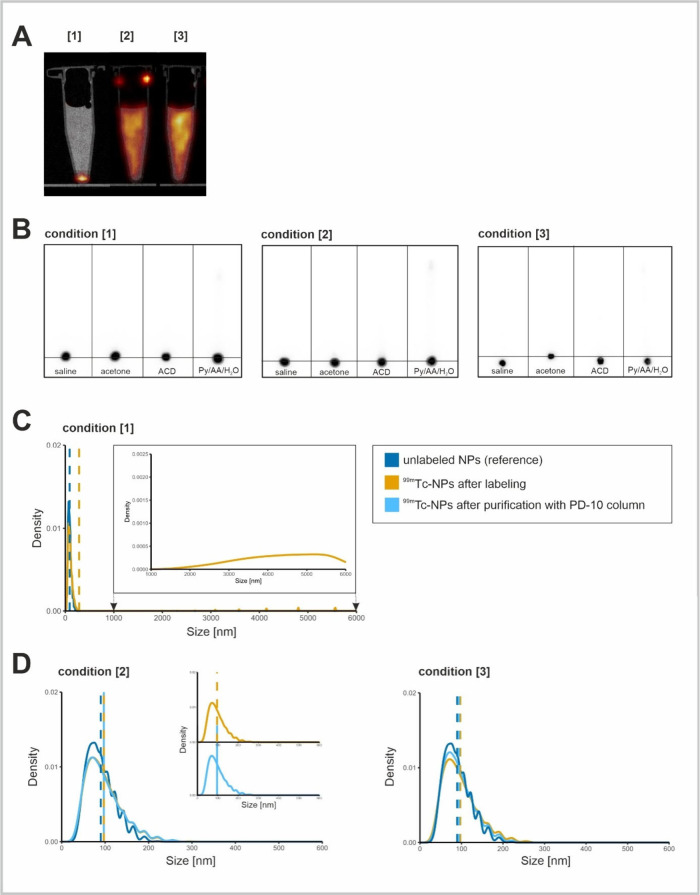
**TLCs and SPECT/CT fusion imaging of NPs radiolabeled under different conditions along with the corresponding density plots of NP size distribution.** (A) SPECT imaging fused with CT of reaction vessels from experimental conditions [1]–[3]. (B) TLCs of the reaction mixtures from labeling conditions [1]–[3]. (C) DLS measurements corresponding to condition [1]. DLS data of unlabeled NPs was plotted as a reference. The mean values were highlighted as vertical dashed lines corresponding to the color of the density curves. (D) DLS measurements corresponding to experimental conditions [2] and [3], before (yellow) and after (light blue) purification with the PD-10 column. *In the case of condition [2], the density curves were additionally displayed separately for better visibility.*

To assess the impact of these findings on radiolabeling and biodistribution, we compared three radiolabeling conditions: a standard reduction with SnCl_2_, an optimized reduction with SnCl_2_, and a reduction with Na_2_S_2_O_4_ (Table 1). The first reaction condition with SnCl_2_ at room temperature was expected to form HR-^99m^Tc (condition [1]). The second condition, still using SnCl_2_ as a reducing agent but at 37 °C for 30 min, was validated for good labeling efficiency (condition [2]). The third one using Na_2_S_2_O_4_ as a reducing agent could prevent the formation of HR-^99m^Tc (condition [3]). Despite all three labeling conditions showing the same results in the radio-TLCs, with no development of sample spots (Rf = 0) in any of the eluent systems described above (Figure 2B), their SPECT imaging demonstrated different characteristics (Figure 2A). In the sample from condition [1], the radioactivity was concentrated at the bottom of the reaction vessel. This observation contradicted the presence of not only free, unbound ^99m^Tc and unreduced ^99m^TcO_4_^–^ but also radiolabeled NPs (89.1 ± 36.2 nm) according to Stokes’ law. DLS measurements further confirmed the presence of colloidal radioactive species adjacent to the unlabeled NPs, detecting colloids in the size range of 2 to 6 μm (*dh*) (Figure 2C, Figure S10). In contrast, samples from conditions [2] and [3] exhibited a homogeneous distribution of radioactivity in the reaction vessels (Figure 2A). Thus, in the current setting, TLC analysis could not contribute to the differentiation of the samples or the characterization of the radioactive species present.

**Table 1:** Overview of the Experimental Conditions and Results

	Reducing agent	Reaction conditions	Expected colloid formation	SPECT imaging	DLS measurements (dh)
[1]	SnCl_2_	RT, 30 min	Yes	clotted radioactivity → colloids	colloids: 1.99–5.56 μm
[2]	SnCl_2_	37 °C, 30 min	No	homogeneously distributed radioactivity	colloids: -
[3]	Na_2_S_2_O_4_	37 °C, 30 min	no mixed colloids (^99m^Tc–Sn(OH)_2_)	homogeneously distributed radioactivity	colloids: -

**Figure 3:**
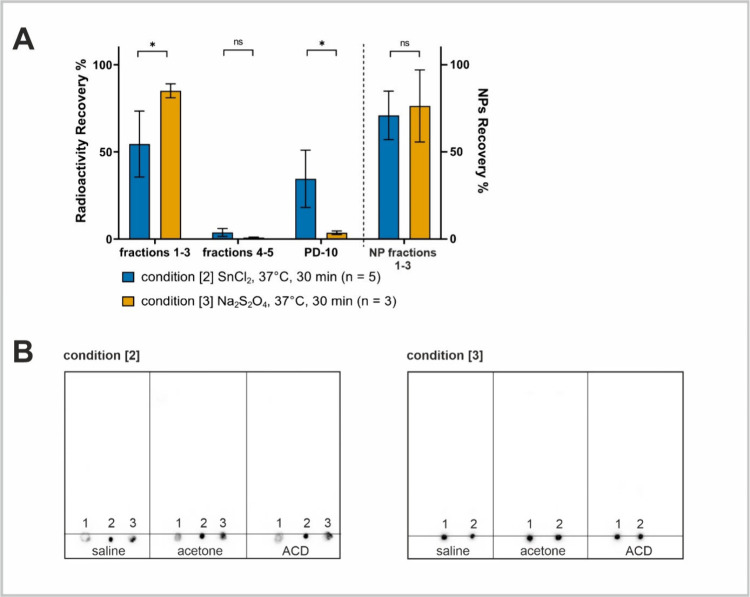
**Characterization of the fractions following purification of reaction mixtures from labeling conditions [2] and [3] via the PD-10 column.** (A) Plot of radioactivity recovery/purification yield (left of dashed line) and nanoparticle recovery/purification yield (right of dashed line) after PD-10 column purification from radiolabeling conditions [2] and [3]. NP recycling yield is calculated by dividing the quantity of the first three collected fractions after PD-10 column purification by the total amount of NPs used for radiolabeling. *P < 0.05, ns = no significance. (B) TLCs of collected fractions after purification from conditions [2] and [3] using saline, acetone, and ACD as developing solvents.

**Figure 4:**
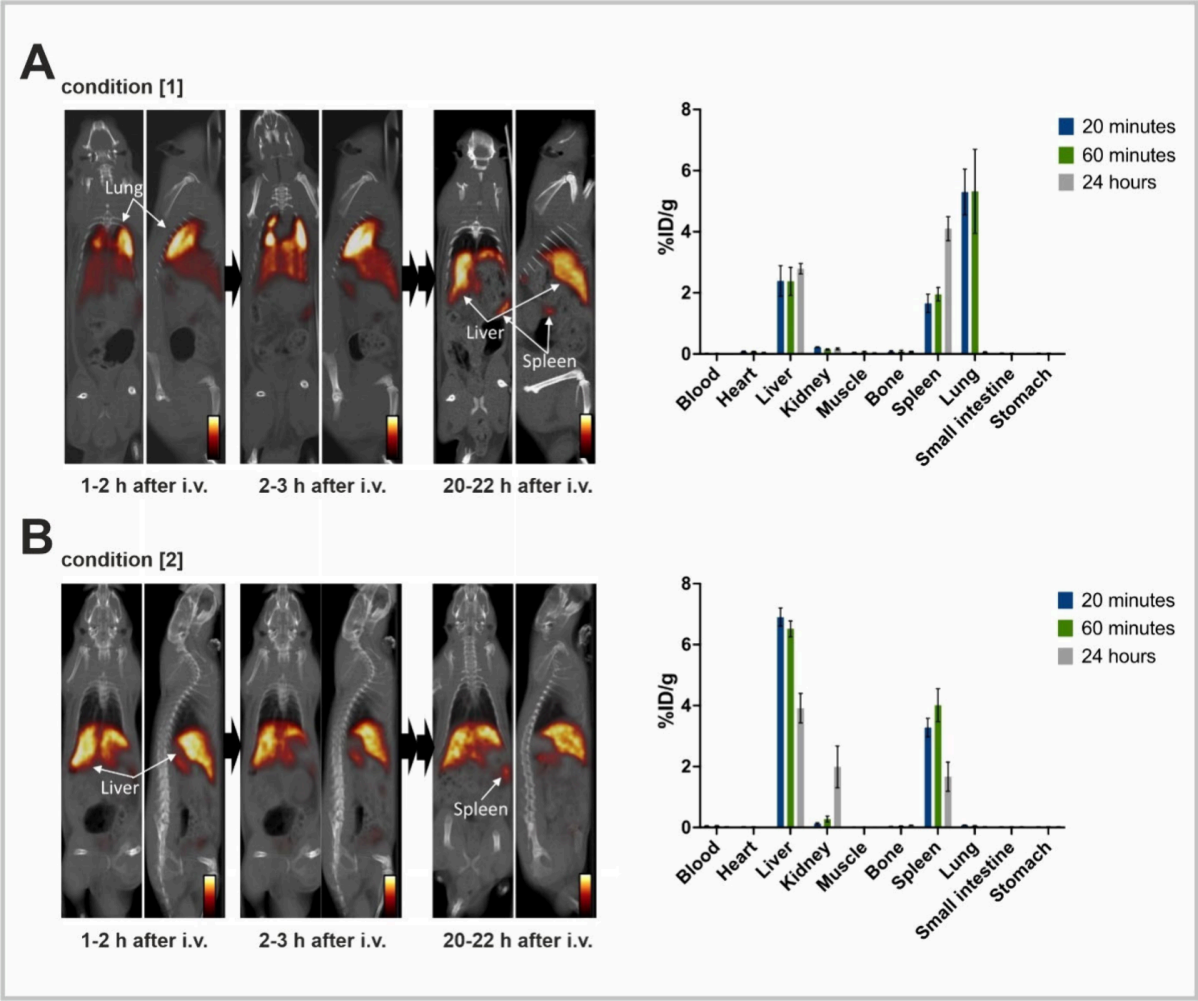
**In vivo studies of**^**99m**^**Tc-NPs.** Dynamic SPECT imaging fused with CT and biodistribution studies (20 min, 60 min, and 24 h) in rats after intravenous administration of ^99m^Tc-NPs radiolabeled under experimental conditions (A) [1] and (B) [2] ([2] after purification with the PD-10 column).

To confirm that the ^99m^Tc in samples from conditions [2] and [3] was indeed bound to the NPs and not present as free species, we subjected the samples to size-exclusion chromatography using a PD-10 column. The first five fractions (1 mL each) were collected and analyzed for radioactivity and NP concentration. Additionally, TEM imaging was performed on all samples to verify that the purification process did not alter the NP morphology (Figure S11). While TLC analysis failed to differentiate the labeling process between these conditions, size-exclusion chromatography indicated higher radiolabeling efficiency of the Na_2_S_2_O_4_-based method over the SnCl_2_ one (Figure 3A). A difference has also been observed in dynamic SPECT/CT imaging and biodistribution studies in rats, after intravenous administration of a sample from condition [1] or [2].^13,14^ The sample prepared under condition [1] favoring HR-^99m^Tc formation exhibited pronounced lung accumulation, followed by liver and spleen due to the large particle size of colloids that is trapped in the capillaries (⌀ 5 μm) in the lungs, consistent with the presence of radioactive impurities that is undetected on TLC (Figure 3B). After 22 h, the radioactivity slowly redistributed and was detected only in the liver and spleen (Figure 4A). In contrast, ^99m^Tc-NPs prepared under optimized condition [2] showed no colloid formation before and after purification according to DLS measurements (Figure 2D, Figure S10) with an expected distribution to the liver, spleen, and kidneys in SPECT/CT imaging in rats (Figure 4B). Overall, the results indicated that the published TLC analysis could not sufficiently differentiate the radioactive species in the present system, which underscores the limitations of TLC for assessing the radiochemical purity of polymeric NPs and emphasizes the critical need for complementary analytical techniques to accurately characterize NP formulations and their radiolabeled counterparts.

## Conclusion

Our findings using PLGA/PLA–PET NPs as a model underscore the limitations of conventional TLC analysis for comprehensively assessing the radiochemical purity of ^99m^Tc-labeled polymer NPs. The complex composition of these NPs, coupled with the challenges associated with analyzing polymeric materials using TLC, hinders the development of universally applicable methods. Furthermore, the potential formation of several possible radioactive impurities including colloids, which can significantly impact in vivo biodistribution, highlights the critical need for robust analytical techniques beyond TLC. To ensure accurate interpretation of in vivo data, a meticulous evaluation and validation of analytical methods are essential for each specific nanoparticle system.

## Experimental Section

### Nanoparticle Preparation

Core–shell nanoparticles were obtained by nanoprecipitation.^24^ For this purpose, PLA_10K_-PEG_5K_-COOH block copolymer was synthesized and poly(d,l-lactide-*co*-glycolide) (PLGA) (Resomer RG 502, acid-terminated, *M*_w_ 7000–17000, Sigma-Aldrich, St. Louis, USA) was covalently labeled with cyanine-5-amine (Lumiprobe GmbH (Europe), Hannover, Germany). CY-5-PLGA as the core material and PLA_10K_-PEG_5K_-COOH for the nanoparticle shell were adjusted in acetonitrile to a concentration of 10 mg mL^–1^, whereby the ratio of core/shell polymer was 3:7 (m/m). The organic phase was added dropwise into the 10-fold excess of stirring (930 rpm) aqueous phase, consisting of 10% (v/v) Dulbecco’s phosphate-buffered saline (DPBS) (Gibco, Life Technologies, Paisly, UK) in Milli-Q water, and stirred for 3 h to remove the acetonitrile. Subsequently, the volume was concentrated by centrifugation at 3000*g* for 15 min using Microseps Advance 30K centrifugal filters (molecular weight cutoff 30 kDa, Pall Corporation, New York, USA). Dynamic light scattering (DLS)-based measurements were carried out with a Malvern Zetasizer Nano ZS instrument (Malvern Instruments, Herrenberg, Germany) to characterize the nanoparticles regarding their size distribution and zetapotential. Nanoparticle concentrations were adjusted based on polymer content [mg mL^–1^], with the amount of polyethylene glycol in each batch, determined using iodine assay, serving as a correlation factor.

### Radiolabeling, TLC Analysis, and Purification

To a mixture of 450 μL NPs (approximately 1 mg mL^–1^) and 50 μL ^99m^TcO_4_^–^ (40–220 MBq in saline) was added 15 μL of freshly prepared SnCl_2_ solution (10 mg mL^–1^ in 0.1 M citric acid or HCl). The pH value of the solution was adjusted to 7–7.5 (Merck accurate pH indicator sticks, pH 5–10), using 0.5 M NaHCO_3_ and 0.5 M Na_2_HPO_4_. In the case of Na_2_S_2_O_4_, 100 μL of freshly prepared Na_2_S_2_O_4_ solution (100 mg mL^–1^ in water) was added to the mixture of NPs and ^99m^TcO_4_^–^, followed by the addition of 0.5 M NaHCO_3_ to a final pH of 5. The solution was mixed gently and allowed to incubate either at room temperature or 37 °C for 30 min. An aliquot of 1 μL was removed afterward and plotted on the starting line of each TLC (Silica gel 60 F254, Merck KGaA, Darmstadt, Germany). Saline, acetone, ACD (citrate dextrose solution, Merck KGaA, Darmstadt, Germany), and pyridine/acetic acid/water 3:5:1.5 (v/v) were used as developing solvent, respectively. The TLCs were exposed to Fujifilm Autoradiography film shortly followed by scanning on an autoradiography system (Amershan Typhoon, GE Healthcare GmbH, Solingen, Germany). Public domain software ImageJ (version 1.53t) was used for quantification. For staining with iodine vapor, the chamber was saturated with iodine vapor and TLC plates were allowed to remain within the chamber until they had developed a light brown color over the entire plate.

For purification of radiolabeled NPs on size-exclusion columns, PD-10 desalting columns (bed volume 8.3 mL) with Sephadex G-25 medium as the fixed phase (GE Healthcare GmbH, Solingen, Germany) were preconditioned by 25 mL of saline. The labeling mixture was loaded to the column, and saline was added to allow the total loading volume to reach 2.5 mL. Saline was used as the mobile phase, and 5 × 1 mL was collected. The radioactivity and NP concentration of each fraction were measured in a dose calibrator and a microplate reader (Hidex Sense Beta Plus, Hidex Deutschland Vertrieb GmbH, Mainz, Germany), respectively. Typical radiochemical yield is calculated by dividing the radioactivity of the first three fractions collected after PD-10 column purification by radioactivity before labeling incubation with decay correction. The NP recycling yield is calculated by dividing the quantity of the first three collected fractions after PD-10 column purification by the total amount of NPs used for radiolabeling.

### SPECT/CT Imaging and Biodistribution Studies

All animal experiments were approved by the Ethics Commission of the government of Lower Franconia, Bavaria, Germany (approval number RUF-55.2.2-2532-2-1765-21) and were conducted strictly according to the Guide for the Care and Use of Laboratory Animals^25^ and the ARRIVE guidelines.^26^ Anesthesia was induced in healthy male Wistar rats (*n* = 1 for each SPECT/CT imaging, *n* = 3 for biodistribution study at each time point, weighing 350–560 g, Charles River Laboratories, Research Models and Services, Japan) by using 5% isoflurane to induce anesthesia and maintained during the whole experiment with 2% isoflurane. SPECT/CT imaging was obtained according to a previous described protocol.^27^ In brief, SPECT imaging in rats was obtained using a dedicated small animal SPECT/CT system (MILabs B.V., Houten, The Netherlands). ^99m^Tc-NPs (100 MBq) were injected via the tail vein of the animals. A SPECT scan was initiated with acquisition in list-mode 1 and 20 h postinjection. The obtained SPECT images were analyzed with the public domain tool AMIDE imaging software (A Medical Imaging Data Examiner, version 1.01). For the biodistribution studies, ^99m^Tc-NPs (0.5 to 1.3 MBq each animal, *n* = 3 each time point) were administered via the tail vein. The animals were euthanized at 20, 60 min, and 24 h after radiotracer administration. The organs of interest were harvested and weighed, followed by tissue counting with a γ-counter (2480 Automatic Gamma Counter WIZARD2, PerkinElmer LAS GmbH, Rodgau, Germany). Results are expressed as a percentage of injected dose per gram of tissue (%ID/g).

## Data Availability

The data will be made available on request.

## Supporting Information Available

Illustration of impurities; radiolabeling studies on CY-5 tagged PLGA/PLA–PEG nanoparticles; reaction mixture of NPs, [TcO_4_^–^], and SnCl_2_ solubilized in citric acid reacted at room temperature for 30 min; extended experimental section (materials; fluorescence labeling of PLGA with CY-5; block copolymer synthesis and characterization; NP characterization; iodine assay; polymer-content-based NP quantification; transmission electron microscopy; in vitro stability and challenge test; calculation of the distance between solvents and polymers in the “Hansen space”; statistics); software; synthesis and characterization of PLGA-CY-5; block copolymer synthesis and characterization; nanoparticle characterization; polymer-content-based NP quantification; DLS raw data; TLCs of polymers and CY-5-amine before and after staining with iodine vapor; Hansen solubility parameters of polymers and solvents and their distances in the “Hansen space”; TEM images; results of in vitro stability and challenge tests; abbreviations (PDF)
