# Supplementary material for: Rethinking Thin-Layer Chromatography for Screening Technetium-99m Radiolabeled Polymer Nanoparticles
Source: ACS Pharmacol Transl Sci. 2024 Aug 30;7(9):2604–11. doi: 10.1021/acsptsci.4c00383 (PMC11406700; doi:10.1021/acsptsci.4c00383)
Supplement: Supplementary file 1 — pt4c00383_si_001.pdf [file pt4c00383_si_001.pdf]

Supplementary information for

Re-thinking Thin-Layer Chromatography for Screening Technetium-99m Radiolabeled Polymer Nanoparticles

Kathrin Schorr[1,#] Xinyu Chen[2,#], Takanori Sasaki[3,4], Anahi Paula Arias-Loza[3], Johannes Lang[1], Takahiro Higuchi[3,4,*], Achim Goepferich[1,*]

[1]Department of Pharmaceutical Technology, University of Regensburg, Regensburg, Bavaria 93053, Germany.

[2]Nuclear Medicine, Faculty of Medicine, University of Augsburg, Augsburg, Bavaria 86156, Germany

[3]Department of Nuclear Medicine and Comprehensive Heart Failure Center, University Hospital Würzburg, Würzburg, Bavaria 97080, Germany

[4]Faculty of Medicine, Dentistry and Pharmaceutical Sciences, Okayama University, Okayama 700-0082, Japan

[#]Equal contribution.

Corresponding auther:

*Achim Goepferich

Email: achim.goepferich@ur.de

Phone: +49 941 943-4842

*Takahiro Higuchi

Email: thiguchi@me.com

Phone: +49 931 201-35455

Author Contributions: A.G., T.H., X.C. and K.S. designed research; X.C. and K.S. performed research; Y.M. and A.P.A. performed the animal studies; J.L. synthesized the polymer; X.C and K.S. analyzed data; A.G., T.H., X.C. and K.S. wrote the paper; A.G. and T. H. supervised the project.

Keywords: polymer nanoparticles, direct ^{99m}Tc-labeling, single-photon emission computed tomography, radio-thin layer chromatography, radiocolloids

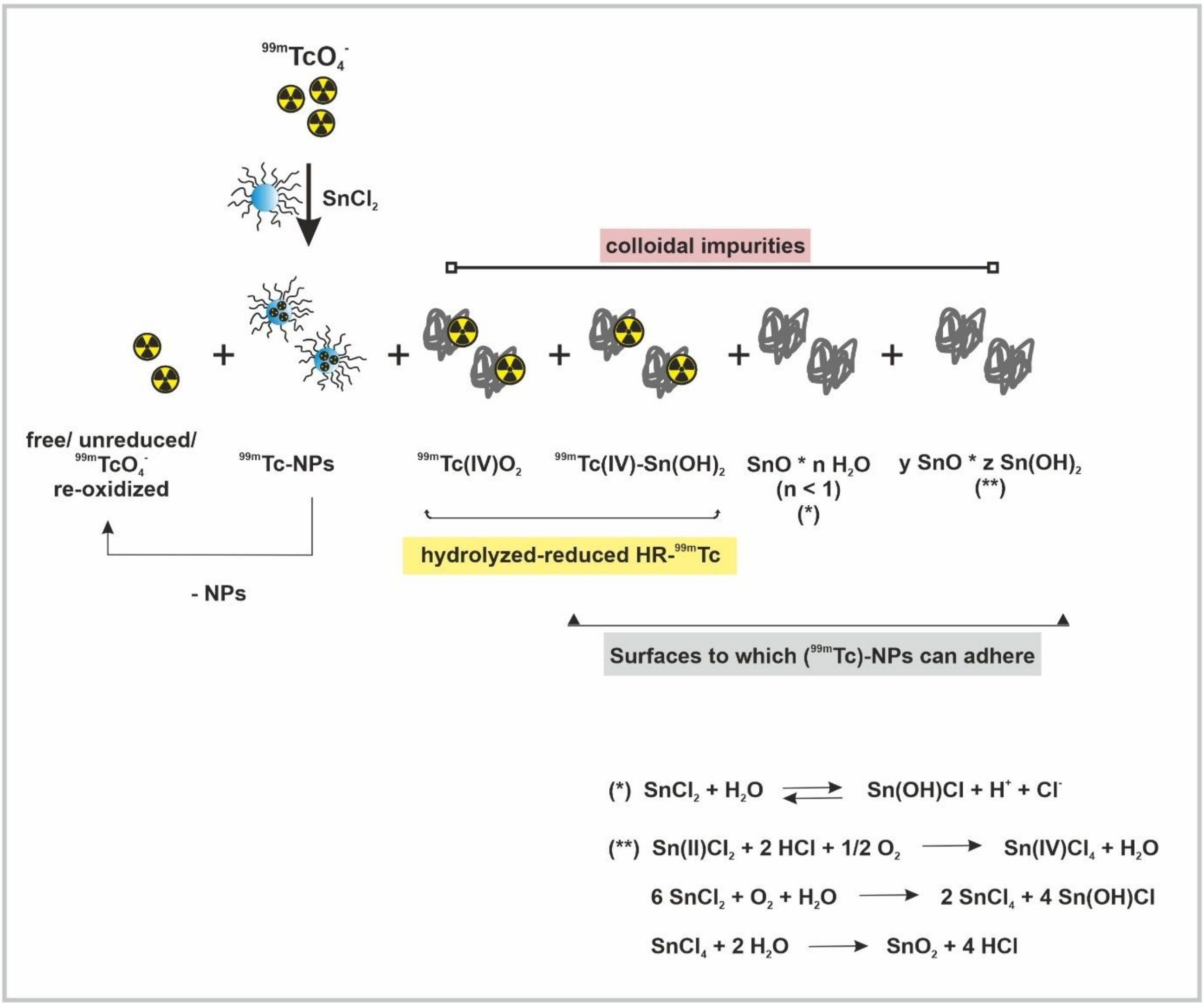

Figure S1. Illustration of impurities. Possible impurities occurring in the reaction vessel[1,2] and underlying tin chemistry. Tin(II) chloride can be hydrolyzed in aqueous solution to an insoluble tin hydroxide salt. In addition to hydrolysis to tin oxide (SnO_2* nH_2O), tin chloride can also be oxidized in the presence of atmospheric oxygen. The resulting tin tetrachloride ($SnCl_4$) is hydrolyzed in aqueous solution[3].

Table A

	Labeling procedure
1)	450 µL of nanoparticles (1.1 mg/ml, pH 5) were added to the reaction vessel
2)	450 µL of [$^{99m}TcO_4$]$^-$ solution in saline were added
3)	A defined volume of $SnCl_2$ solution (2 mg/ml, solubilized in 10 mM HCl)<br>**[1]** 100 µL $SnCl_2$ solution was added **[2]** 20 µL $SnCl_2$ solution was added
4)	The reaction mixture was stirred at 500 rpm for 1.5-2 h at room temperature

A

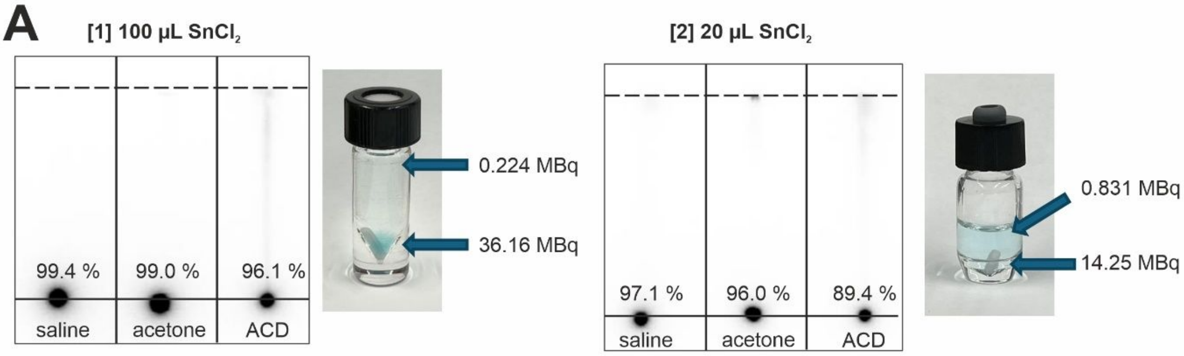

Table B

	Labeling procedure
1)	450 µL of [$^{99m}TcO_4$]$^-$ solution in saline were added to the reaction vessel
2)	Vacuum was applied to the reaction vessel and it was subsequently purged with nitrogen
3)	A defined volume of $SnCl_2$ solution (1 mg/ml, solubilized in 100 mM HCl)<br>**[3]** 60 µL $SnCl_2$ solution was added **[4]** 100 µL $SnCl_2$ solution was added
4)	The reaction mixture was stirred at 500 rpm for 5 min at room temperature
5)	10 µL of 0.5 M $NaHCO_3$ solution and 450 µL of nanoparticles (1.1 mg/ml) were added
6)	The reaction mixture was stirred at 500 rpm for 20 min at room temperature

C

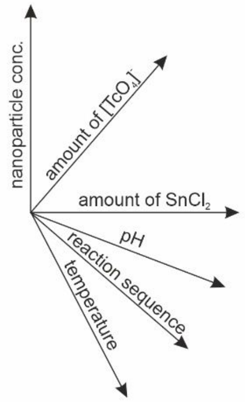

B

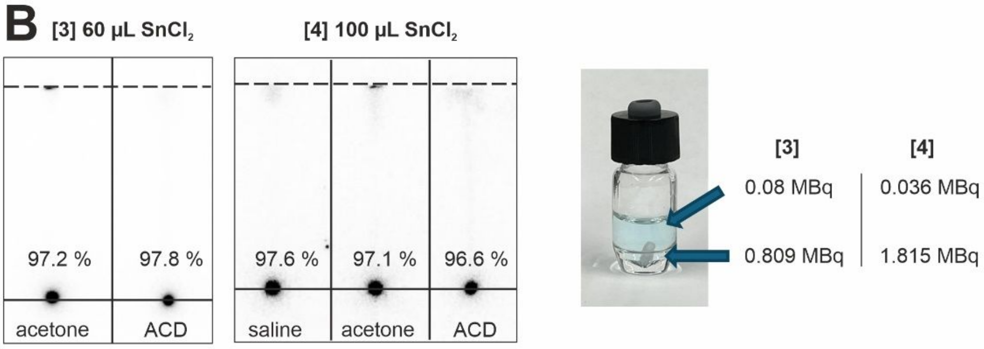

Figure S2. Radiolabeling studies on CY-5 tagged PLGA/PLA-PEG nanoparticles. (Table A-B) Exemplary representation of labeling protocols with $^{99m}TcO_4^-$ and $SnCl_2$ as reducing agent, and (A-B) the resulting batches and their TLC analysis using saline, acetone and ACD solution as developing solvents. (C) Main factors influencing the labeling efficiency of nanoparticles[4–8].

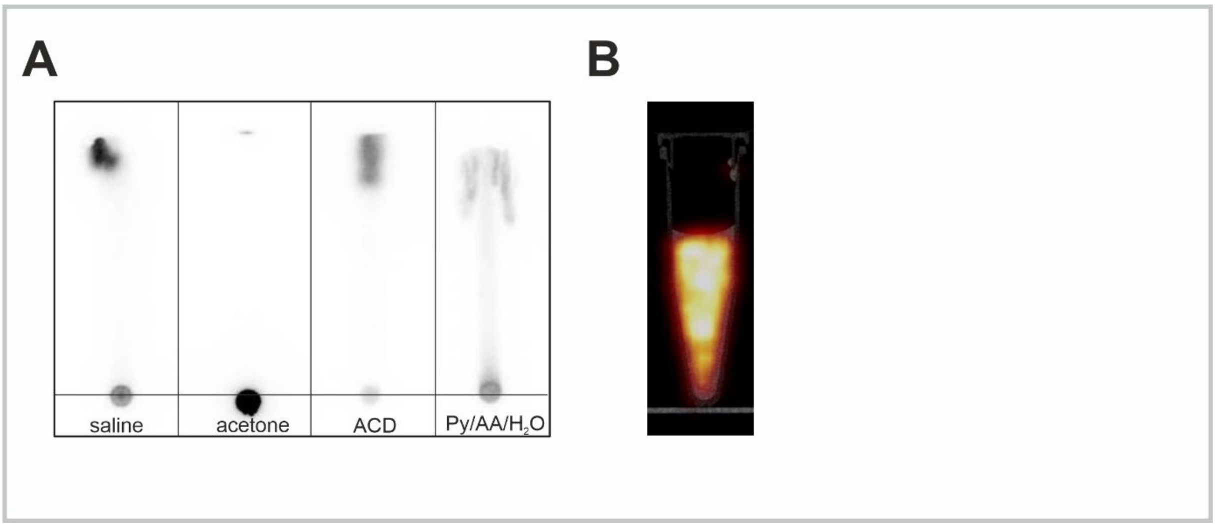

Figure S3. Reaction mixture of NPs, $[TcO_4^-]$ and $SnCl_2$ solubilized in citric acid reacted at room temperature for 30 min. (A) TLCs of the reaction mixture. Saline, acetone, ACD and Py/AA/H_2O were used as developing solvents. (B) SPECT/CT fusion imaging of the reaction mixture.

Extended experimental Section

Materials

All materials and reagents described below were purchased from Sigma Aldrich (Taufkirchen, Germany or St. Louis, USA) unless otherwise stated. The ultrapure water used for the experiments was treated with a Milli-Q EQ 7000 system (Merck, Darmstadt, Germany) equipped with Milli-Q Biopak filter (Merck, Darmstadt, Germany) and taken fresh every day. It is referred to as Milli-Q water in the following.

Fluorescence labeling of PLGA with CY-5

Poly(D,L-lactide-co-glycolide) (PLGA) (Resomer RG 502, carboxylic acid-terminated, M_w 7000-17000, Sigma-Aldrich, St. Louis, USA) was fluorescently labeled by covalent linkage with cyanine-5-amine (Lumiprobe GmbH (Europe), Hannover, Germany). In brief, carboxylic acid-terminated PLGA (1 equiv.) was dissolved together with cyanine-5-amine (0.1 equiv.) in anhydrous *N,N*-dimethylformamide (DMF). The carboxylic acid group was activated by the addition of (2-(1H-benzotriazol-1-yl)-1,1,3,3-tetramethyluronium hexafluorophosphate (HBTU) (2 equiv.) and *N,N*-diisopropylethylamine (DIPEA) (4 equiv.) and the reaction mixture was stirred overnight at room temperature under light protection. To remove the excess of free dye, the polymer was precipitated several times in ice-cold diethyl ether until the supernatant was no longer blue. The product was dried under the nitrogen flow and characterized by FTIR. FTIR spectra were recorded on an Agilent Technologies Cary 630 FTIR (Agilent, Santa Clara, USA) with 32 scans, 8 cm^{-1} resolution. (Figure S4) The purity was confirmed by high performance liquid chromatography (HPLC) analysis (Agilent Technologies 1260 Infinity II, Santa Clara, USA). For this, a C18 column (Kinetex 2.6 µm, EVO C18 100 Å, 150 x 4.6 mm) was used, that was heated to 40 °C. 0.05 % trifluoroacetic acid in acetonitrile [A] and 0.05 % trifluoroacetic acid in Milli-Q water [B] were employed as the mobile phase for the gradient elution at a flow rate of 1.0 mL min^{-1} ([A]10 to 95 % - 0-10 min/ [A] at 95 % - 10-13 min/ [A] 95 to 10 % - 13-15 min/ [A] at 10 % - 15-20 min). Fluorescence detection was performed at excitation λ 647 nm and emission λ 665 nm. Emission spectra were recorded in the peaks (scan range: λ 630 nm to 680 nm, step: 5 nm). 2 µL sample volume (dissolved in acetonitrile, polymers diluted to a concentration of 3.3 µM, CY-5-amine diluted to a concentration of 0.2 µM) were applied in each run. Additionally, PLGA-CY-5 polymer nanoparticles were prepared in Milli-Q water from the analyzed polymer batch and purified with a PD-10 column (Sephadex™ G-25M, cytiva, Buckinghamshire, UK) to double-prove the elution behavior of polymer free from unreacted CY-5-amine and polymer fragments. After purification the samples were freeze-dried (Alpha 2-4 LSCplus/ Christ/ Osterode am Harz/ Germany), dissolved in DCM and TLC analysis was performed.

Block copolymer synthesis and characterization

COOH-PEG_{5k}-PLA_{10k} block copolymer was synthesized via ring-opening polymerization of the cyclic lactone 3,6-dimethyl-1,4-dioxane-2,5-dione with the organocatalyst 1,8-diazabicyclo[5.4.0]undec-7-ene (DBU) at room temperature. COOH-PEG_{5k}-OH (Jenkem Technology, Plano, USA, M_n = 5000 ± 500 g mol^{-1}, as stated on certificate of analysis) is used as macroinitiators for the reaction[9,10]. In brief, polyethylene glycol (PEG) macroinitiator (1 equiv.) is reacted with 3,6-dimethyl-1,4-dioxane-2,5-dione (70 equiv.) and DBU (3.5 equiv.) in dichloromethane (DCM) as solvent at room temperature. After 1 h the reaction is quenched with benzoic acid in excess (17.5 equiv.) and the resulting block copolymer is precipitated in ice-cold diethyl ether, filtered, and dried under the nitrogen flow. Identity and purity of the block copolymer was verified by *^1H*-NMR spectra recorded on a Bruker Avance III HD 400 operating at 400 MHz; the block copolymer was dissolved in deuterated chloroform ($CDCl_3$)[11]. The purity was also confirmed by high performance liquid chromatography (HPLC) analysis (Agilent Technologies 1260 Infinity II, Santa Clara, USA). For this, a C18 column (Kinetex 2.6 µm, EVO C18 100 Å, 150 x 4.6 mm) was used, that was heated to 40 °C. 0.05 % trifluoroacetic acid in acetonitrile [A] and 0.05 % trifluoroacetic acid in Milli-Q water [B] were employed as the mobile phase for the gradient elution at a flow rate of 1.0 mL min^{-1} ([A]10 to 95 % - 0-10 min/ [A] at 95 % - 10-13 min/ [A] 95 to 10 % - 13-15 min/ [A] at 10 % - 15-20 min). Fluorescence detection was performed at excitation λ 647 nm and emission λ 665 nm. Emission spectra were recorded in the peaks (scan range: λ 630 nm to 680 nm, step: 5 nm). 2 µL sample volume (dissolved in acetonitrile, polymers diluted to a

concentration of 3.3 µM, CY-5-amine diluted to a concentration of 0.2 µM) were applied in each run. Additionally, PLGA-CY-5 polymer nanoparticles were prepared in Milli-Q water from the analyzed polymer batch and purified with a PD-10 column (Sephadex™ G-25M, cytiva, Buckinghamshire, UK) to double-prove the elution behavior of polymer free from unreacted CY-5-amine and polymer fragments. After purification the samples were freeze-dried (Alpha 2-4 LSCplus/ Christ/ Osterode am Harz/ Germany), dissolved in DCM and TLC analysis was performed. Furthermore, the molecular weight of the synthesized polymer was determined from the *¹H*-NMR spectra as previously described by *Zimmer and Goepferich*[12]. Topspin 4.3.0 software was used to evaluate the NMR spectra. (Figure S5)

NP characterization

Dynamic light scattering (DLS)-based measurements (particle size distribution, zeta potential, stability data) were carried out with a Malvern Zetasizer Nano ZS (Malvern Instruments, Herrenberg, Germany) equipped with a 633 nm He-Ne laser operating at an angle of 173 °C. The software used for data collecting and processing was Zetasizer software version 7.12 (Malvern Instruments, UK). To determine the size distribution, the concentrated samples were diluted 1:20 with 10 % DPBS in Milli-Q water (10 % Dulbecco´s phosphate-buffered saline (DPBS) (v/v)). 90 µL sample volume were measured in micro-UV-cuvettes (Carl Roth, Karlsruhe, Germany) at a controlled temperature of 25 °C (120 sec equilibration time). Three repetitions with 11 runs of 10 sec duration were performed for each sample. The data was analyzed with the software-integrated analysis model for "general purposes (normal resolution)". For zeta potential measurements the samples were diluted 1:200 with 10 % PBS in Milli-Q water. Samples were measured in folded capillary zeta cells (Malvern, Herrenberg, Germany) at 25 °C. Three repetitions with at least 60 runs were performed for each sample. The data was analyzed by "auto mode". (Figure S6)

Iodine Assay

The PEG amount was quantified via an iodine assay adapted from Childs[13] as previously published by *Abstiens and coworkers*[9]. In brief, poly(ethylene glycol) monomethyl ether with an average molecular weight of 5000 g mol^{-1} (mPEG5k) was used as calibration standard in aqueous solutions (0 – 20 µg mL^{-1}). 140 µL of standard dilutions and nanoparticles as samples were pipetted into a 96-well plate and incubated for 10 min under light exclusion with 60 µL of a solution composed of two parts of 5 % barium chloride (m/V) (Merck, Darmstadt, Germany) dissolved in 1 M HCl and one part 0.05 M iodine solution (VWR, Lueven, Belgium). The absorbance was measured at λ 535 nm on a FLUOstar Omega plate-reader (BMG Labtech, Ortenberg, Germany). (Figure S7)

Polymer content-based NP quantification

To determine the nanoparticle concentration in units of [mg mL^{-1}] based on polymer content, three independent batches of nanoparticles were prepared in pure Milli-Q water according to the procedure described for NP preparation. The nanoparticles´ PEG content was determined via colorimetric iodine assay[13]. Furthermore, 100 µL of each nanoparticle batch were freeze-dried (Alpha 2-4 LSCplus, Christ, Osterode am Harz, Germany) and the remaining dry polymer was accurately weighed to determine the mass-concentration [mg mL^{-1}] (mass-based quantification). Assuming constant amounts of PEG between batches of the same type of nanoparticles, the mass concentration was correlated with the PEG content, using the correlation factor *k* as previously described by *Abstiens and coworkers*[9]. (Figure S7)

This approach allowed for subsequent mass-based quantification of the nanoparticles on the basis of the determined PEG content as shown in eq. 1.

$$c(NP)\left[\frac{mg}{ml}\right] = c\ (PEG)\left[\frac{\mu g}{ml}\right] \times k$$

eq. 1

Transmission electron microscopy

For sample preparation 9 nm carbon film coated onto 3.5 nm Cu TEM grids (400 mesh) (Plano GmbH, Wetzlar, Germany) were hydrophilized in the oxygen plasma and 5 µL of the respective nanoparticle dispersion were applied to the grids for 5 sec and in the last application cycle for 1 min, up to four times depending on the nanoparticle concentration in the dispersion. Afterwards, the excess samples were removed carefully with a filter paper, and the copper-grids were washed with Milli-Q water and exposed for 30 s to an aqueous dilution of uranyl acetate (1 %). A stack of images at different positions was recorded on JEM-2100F Field emission electron microscope (Jeol, Freising, Germany) at 20,000-fold magnification using the SerialEM software. The images were analyzed using Fiji[14] software. (Figure S11)

In vitro stability and challenge tests

The stability of ^{99m}Tc-NPs was assessed both in vitro, in saline, and in co-incubation with human serum. Specifically, 100 µL of the freshly collected fraction after PD-10 purification was combined with an equal volume of either saline or human serum, followed by an incubation at 37°C for 1-4 hours. Challenge tests were conducted similarly, with 100 µL of the freshly collected fraction after PD-10 purification being incubated with 10 mM DTPA or cysteine solution at 37°C for 1-4 hours. At each hour interval, an aliquot was withdrawn and subjected to TLC analysis using saline, acetone, and ACD solution as the developing solvents. Subsequently, the TLC plates were examined using an autoradiography system, following the aforementioned protocol. (Figure S12)

Calculation of the distance between solvents and polymers in the “Hansen space”

The solubility of the polymeric components of the nanoparticles in the solvents used as mobile phase for TLC analysis was estimated based on their Hansen solubility parameters. The concept of solubility parameter (δ) was first introduced by Hildebrand, defined as square root of the quotient of cohesion energy (ΔH_v) and the molar volume (V_M)[15], as shown in equation 2:

$$\delta = \left(\frac{\Delta H_v - RT}{V_M}\right)^{1/2}$$

eq. 2

The concept was later expanded by splitting the Hildebrand solubility parameter into three solubility parameters derived from different types of cohesive forces by Hansen:

$$\delta = (\delta_d^2 + \delta_p^2 + \delta_h^2)$$

eq. 3

δ_d, referred to as dispersion parameter, corresponds to the London interactions that the analyzed substance can enter, δ_p, referred to as polar parameter, correlates with the polar groups, and δ_h, referred to as hydrogen bonding parameter, represents hydrogen bonding forces. Solvents and solutes with similar Hansen solubility parameters are expected to form thermodynamically more stable solutions[15].

The solubility of the polymers in the used solvents can therefore be estimated by calculating their distance (D) in the so-called “Hansen space” according to equation 4, where the parameters of the solvents are denoted by S and the parameters of the polymers by P:

$$D = \left(4 * (\delta_{dS} - \delta_{dP})^2 + \left(\delta_{pS} - \delta_{pP}\right)^2 + (\delta_{hS} - \delta_{hP})^2\right)^{1/2}$$

eq. 4

The parameters and their distances are listed in (Figure S9). The following simplifications were made:

(1) The PLA-PEG block copolymer was split into PLA and PEG.

(2) PLGA was considered without the covalently coupled CY-5.
(3) The solvents were not analyzed as a mixture.

Statistics

Results in collected fractions after PD-10 column purification are displayed as the mean ± SD (Figure 3). Multiple group comparisons were performed using analysis of variance (two-way ANOVA). A *P* value of less than 0.05 was assumed to be statistically significant. Statistical analysis was performed with GraphPad Prism (version 9.4.1 GraphPad Software, San Diego, USA).

Software

The software used for the different experiments is listed below.

TopSpin 4.3.0 software was used for NMR processing and analysis.

Origin 2024 software (OriginLab Corporation/ Northampton/ Ma/ USA) for data plotting and analyzing.

CorelDRAW 2023 Graphics Suite was used for graphical illustrations.

R software (R Core Team (2022). R: A language and environment for statistical computing. R Foundation for Statistical Computing, Vienna, Austria. URL hhttps://www.R-project.org/) for data plotting.

Fiji software[14] for TEM image processing.

PerkinElmer ChemOffice Suite 2018 was used to draw chemical structural formula and chemical reaction equations.

Public domain software ImageJ (version 1.53t) was used for the quantitative evaluation of the TLC analysis.

Prism GraphPad (version 19.4.1) was used for the plotting and statical analysis of elution fractions after PD-10 purification.

AMIDE imaging software (A Medical Imaging Data Examiner, version 1.01) was used to analyze the SPECT images.

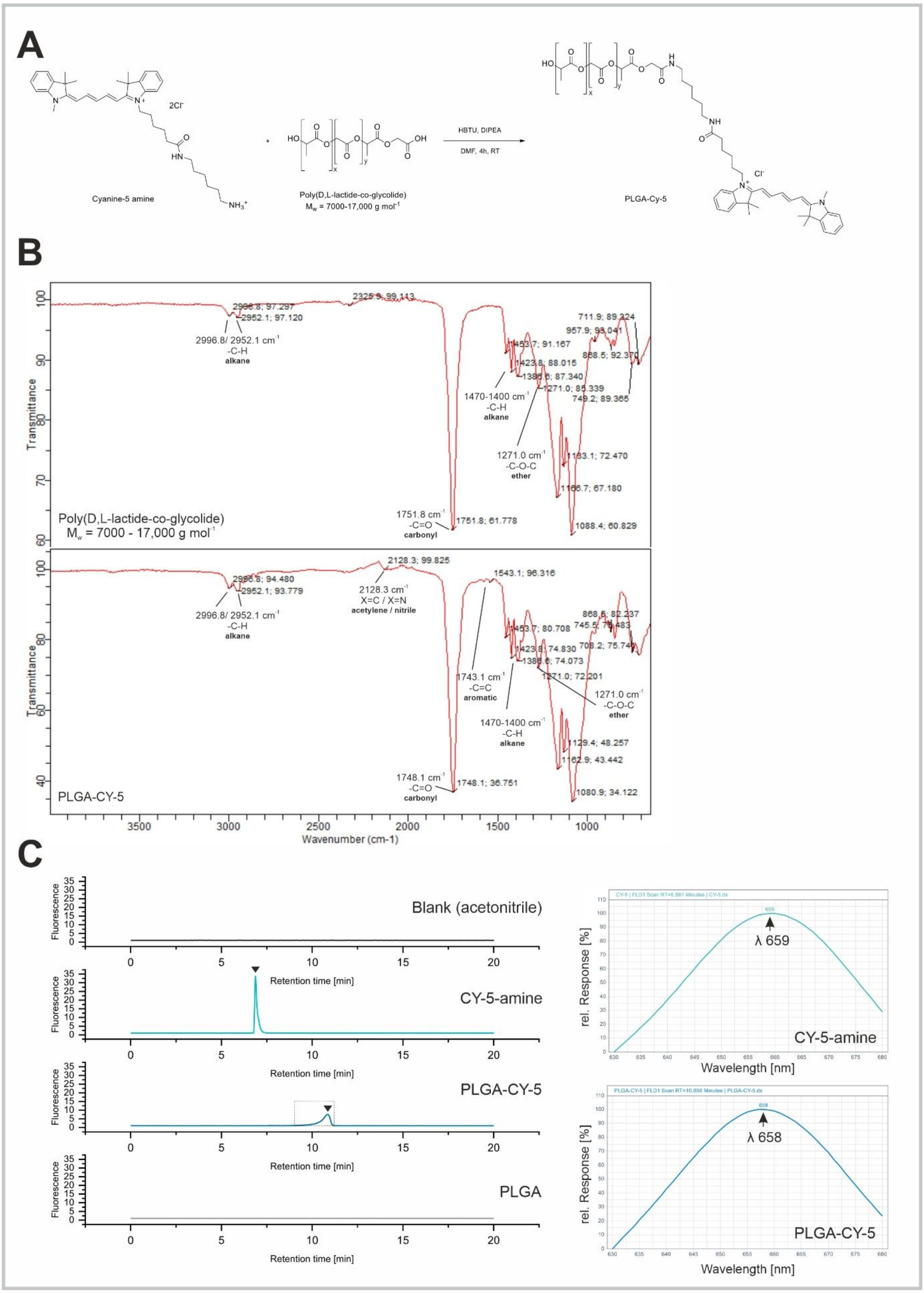

Figure S4. Synthesis and characterization of PLGA-CY-5. (A) Reaction equation of fluorescence labeling of PLGA with CY-5. (B) FTIR spectra of poly(*D,L*-lactide-co-glycolide) (PLGA, M_w = 7000-17,000 g mol^{-1}) and PLGA-CY-5. Peaks for acetylene/ nitrile (2128.3 [cm^{-1}] ν (X=C/ X=N)) and aromatic groups (1743.1 [cm^{-1}] ν (-C=C)) were found in the spectrum of PLGA-CY-5. (C) HPLC chromatograms of acetonitrile (blank), CY-5-amine (sample), PLGA-CY-5 (sample) and PLGA (reference) and emission

spectra of the peaks (CY-5-amine: 6.879 min (RT) / PLGA-CY-5: 10.856 min (RT)) to prove the purity of the CY-5-labeled polymer from unreacted CY-5-amine and that the dye was bound to the polymer. The width (RT 8.5-11.5 min) of the PLGA-CY-5 peak serves as good indicator for the broad molecular weight distribution of the polymer (M_w 7000-17000) and the varying retention times depending on this.

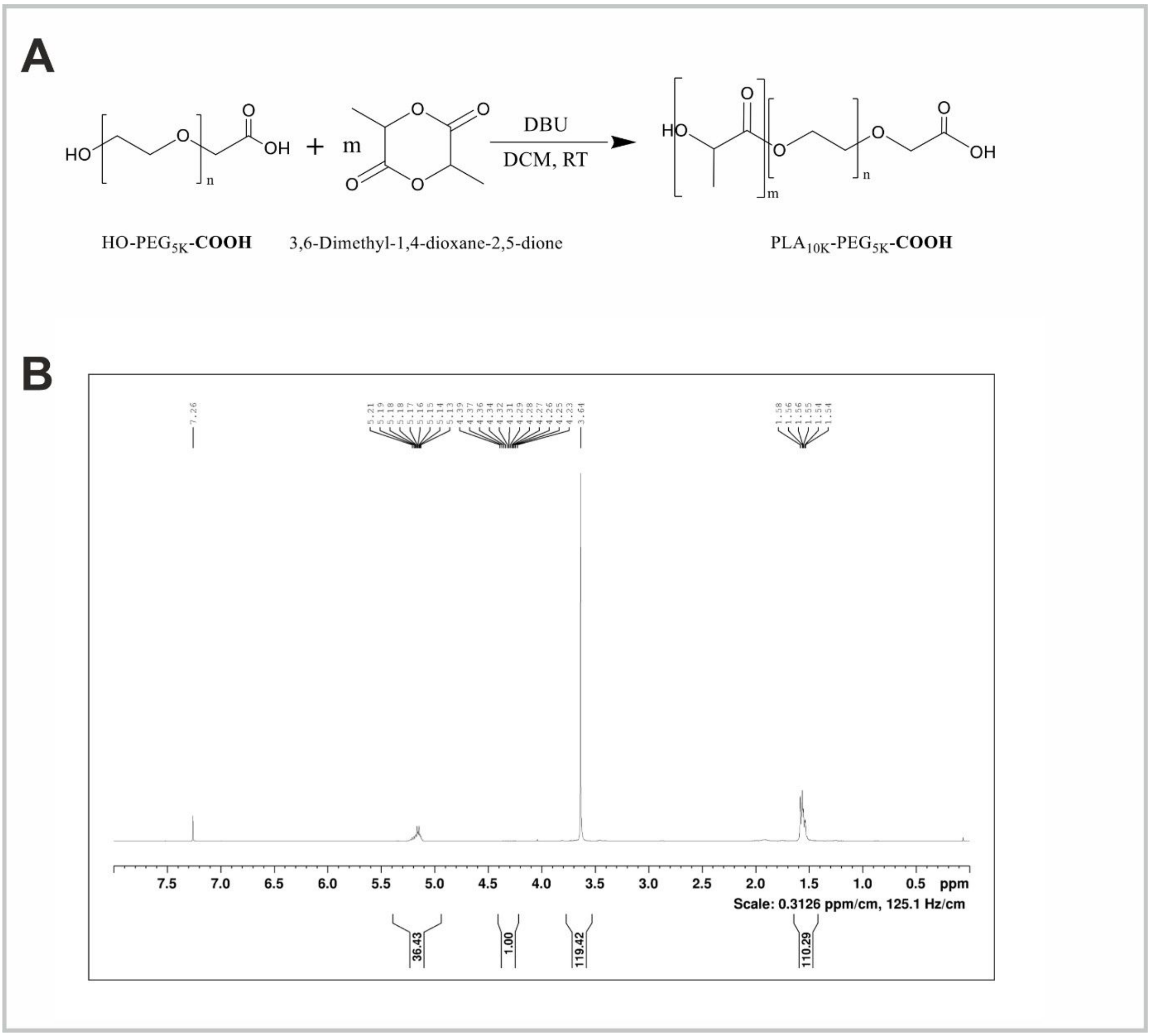

Figure S5. Block copolymer synthesis and characterization. (A) Reaction equation for the synthesis of PLA_{10K}-PEG_{5K}-COOH block copolymer through ring opening polymerization of cyclic lactone 3,6-dimethyl-1,4-dioxane-2,5-dione with polyethylene glycol chains as macroinitiators for the reaction. (B) ^{1}H-NMR spectrum ($CDCl_3$, 400 MHz) of PLA_{10K}-PEG_{5K}-COOH block copolymer. **δ (ppm):** 5.17 ppm (m, -C(C**H_3**)H-), 4.31 ppm (m, -OCH_2CH_2-O(CO)-), 3.64 ppm (m, $HOOCCH_2CH_2$-), 1.56 ppm (m,-C(CH_3)**H**-).

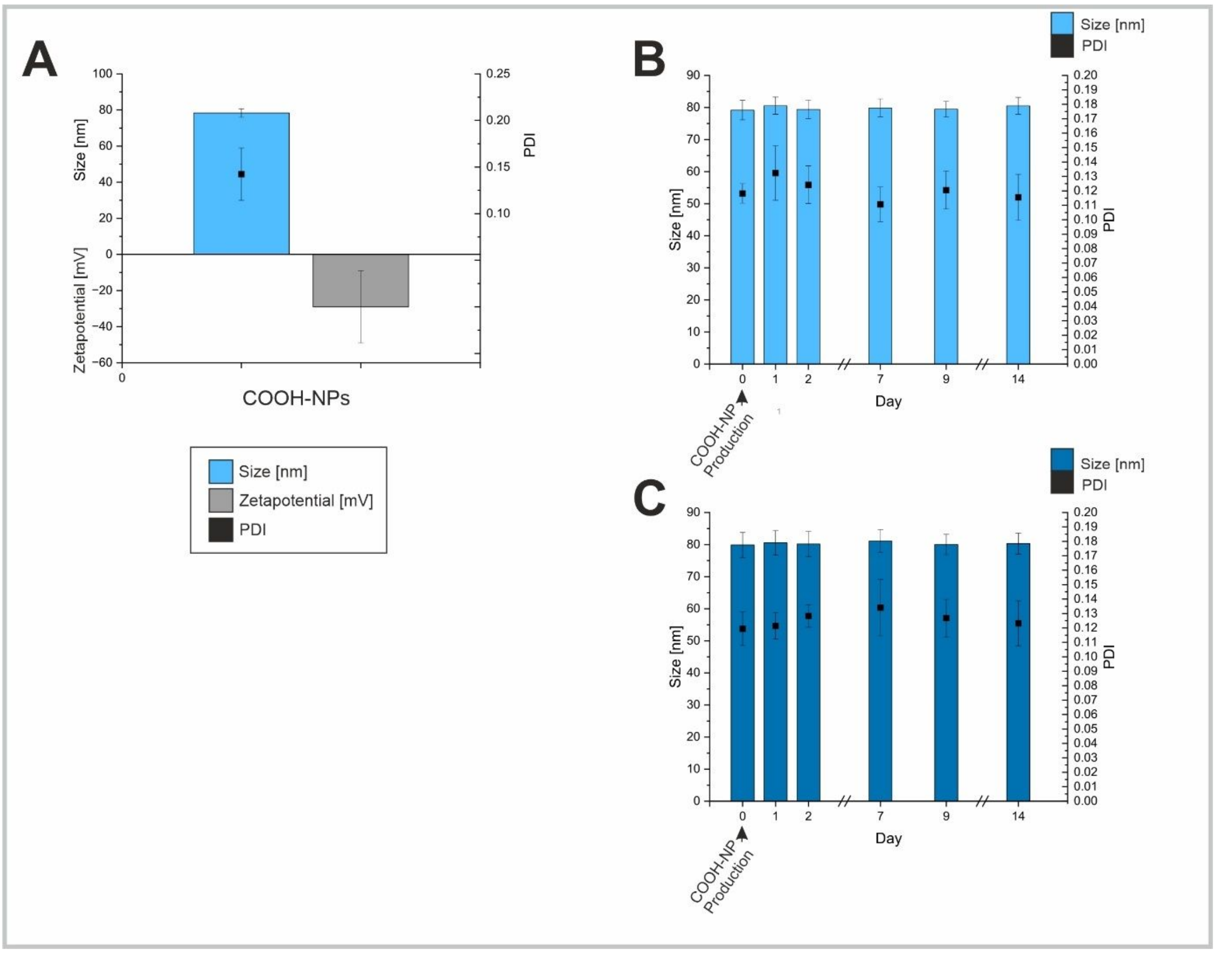

Figure S6. Nanoparticle characterization. (A) Nanoparticle data on hydrodynamic diameters, polydispersity index (PDI) and zeta potential obtained from DLS measurements. Results represent mean ± SD (N = 3; n = 3). (B) Stability data (hydrodynamic diameter and PDI) of COOH-NPs at 22 °C over 14 days. Results represent mean ± SD (N = 3; n = 3). (C) Stability data of COOH-NPs at 2-8 °C over 14 days. Results represent mean ± SD (N = 3; n = 3). The data indicate that the nanoparticles were stable in size over 14 days under both storage conditions.

A

Batch	Size [nm] (mean ± SD) (DLS)	PDI (mean ± SD)	Mass-based quantification<br>conc. [mg mL^{-1}]	Implementation of PEG-conc. as correlation factor (k) determined by iodine assay<br>PEG-conc. [µg/ml]	k
1	83.99 ± 0.07	0.085 ± 0.011	24.8	3862.6	0.00642
2	85.64 ± 0.76	0.082 ± 0.007	25.7	4150.8	0.00619
3	77.96 ± 0.81	0.086 ± 0.002	20.2	2873.2	0.00703
					mean: 0.00655

B

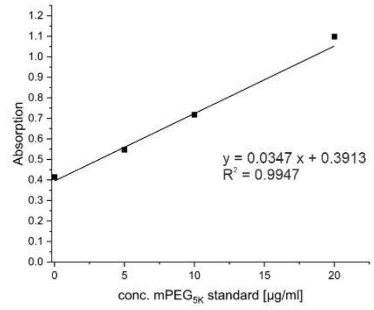

Figure S7. Polymer content-based NP quantification. (A) Production and characterization of three independent nanoparticle batches (COOH-NPs) in pure Milli-Q water to determine correlation factors *(k)* and calculate polymer content-based NP concentrations. Results represent mean ± SD (n = 3). (B) Calibration curve of the iodine assay against $mPEG_{5K}$ standard. Results represent mean ± SD (n = 3).

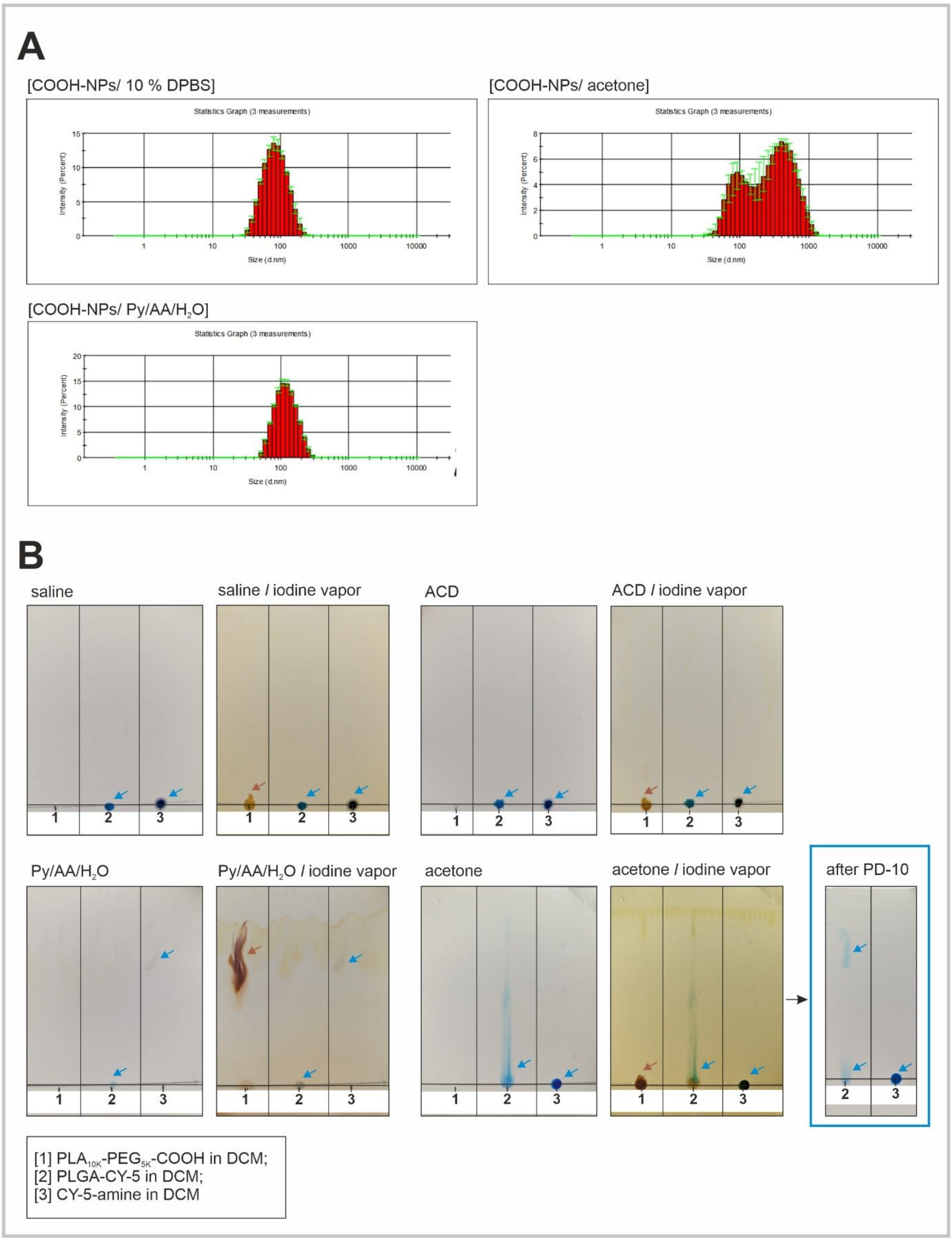

Figure S8. (A) DLS raw data recorded with a Malvern Zetasizer Nano ZS (n = 3). COOH-NPs/ 10 % DPBS: sample of unlabeled NPs diluted 1:20 with 10 % DPBS in Milli-Q water as reference. COOH-NPs/ acetone: sample of unlabeled NPs diluted 1:4 with acetone and subsequently diluted 1:20 with 10 % DPBS in Milli-Q water, following the aforementioned protocol. COOH-NPs/ Py/AA/H_2O: sample of unlabeled NPs diluted 1:4 with a mixture of pyridine: acetic acid: water (3:5:1.5, (v/v)) and subsequently diluted 1:20 with 10 % DPBS in Milli-Q water, following the protocol. (**B) TLCs of polymers and CY-5-amine before and after staining with iodine vapor.** [3] CY-5-amine served as control. The TLC analysis with acetone as solvent was repeated after purification of the PLGA-CY-5 with a PD-10 column to exclude the possibility that the polymer sample was contaminated with unreacted CY-5-amine or

polymer fragments (lactides). The elution behavior of the polymer did not differ before and after purification.

Table A

Component	δ_d	δ_p	δ_h
**PEG (#1)**	20.3	9.6	6.0
**PLA (#1)**	19.8	4.0	6.7
**PLGA (#1)**	17.4	9.1	10.5
**acetonitrile (#2)**	15.3	18.0	6.1
**acetone (#3)**	15.7	5.3	11.7
**pyridine (#2)**	19.0	8.8	5.9
**acetic acid (#3)**	14.5	8.0	13.5

Table B

	Distance D		
	**PEG**	**PLA**	**PLGA**
**acetonitrile**	13.1	16.7	10.8
**acetone**	11.6	9.7	5.2
**pyridine**	2.7	5.1	5.6
**acetic acid**	13.9	13.2	6.6

B

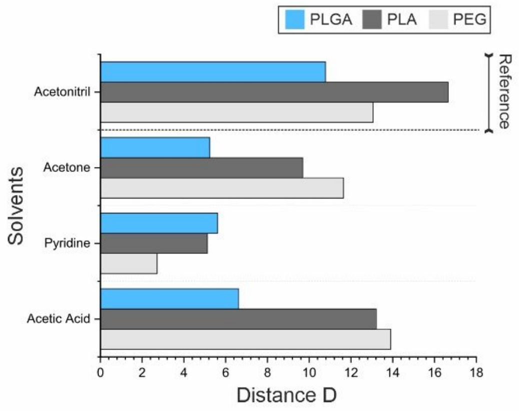

Figure S9. Hansen solubility parameters of polymers and solvents and their distances in the "Hansen space". (#1 obtained from *Maslanka Figueroa, Fleischmann et al. 2020*[16], #2 obtained from *Tang, Zhang et al. 2015*[17], #3 obtained from *Bordes, Fréville et al. 2010*[15]). (B) The solubility of the individual polymers in the mobile phase was calculated as an estimate of the distance D between solvent and solute in the "Hansen space". In direct comparison to acetonitrile, which was used as organic phase in the nanoparticle production process, the polymers showed comparable or better solubility in the organic solvents and acetic acid.

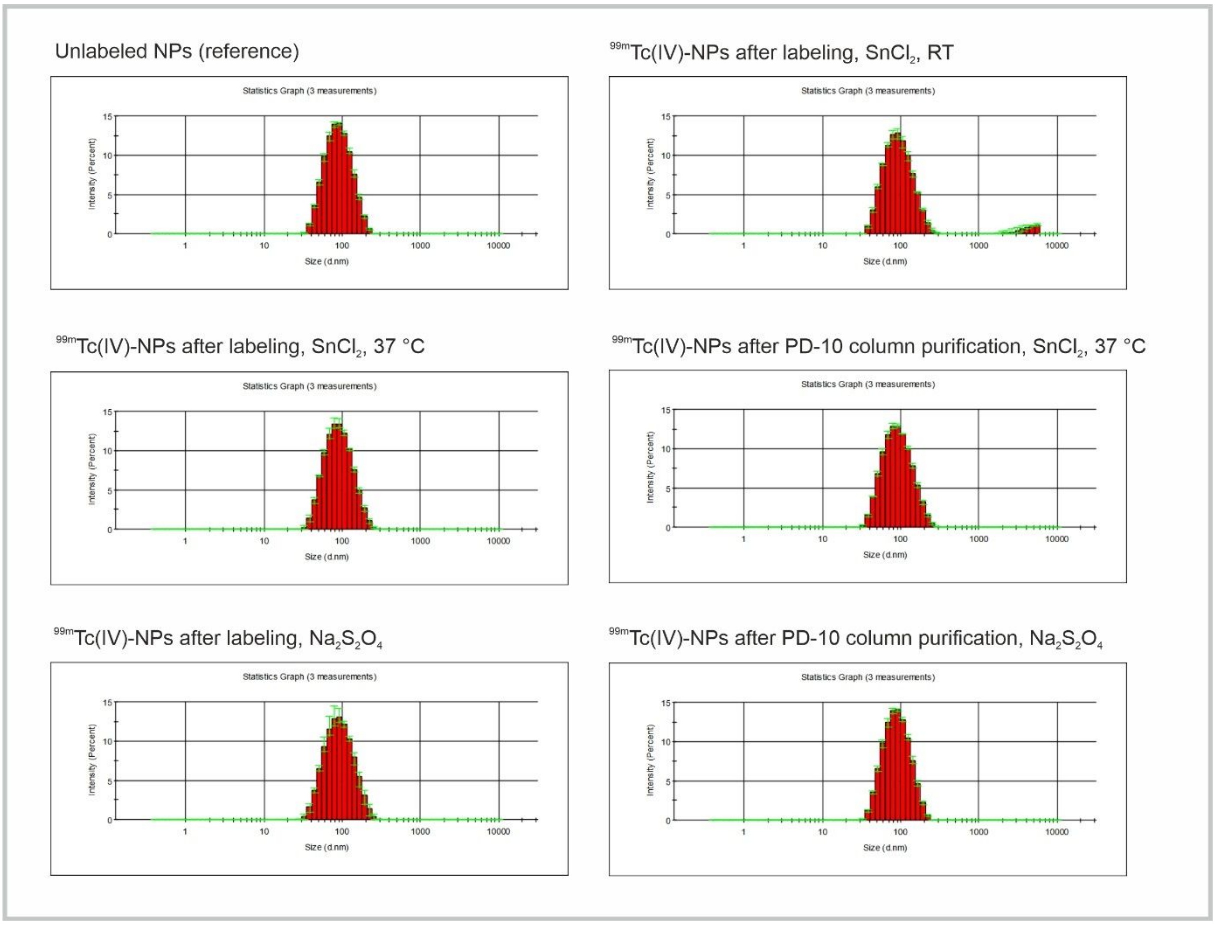

Figure S10. DLS raw data recorded with a Malvern Zetasizer Nano ZS (n = 3). Representative measurements of a sample of NPs before labeling with ^{99m}Tc(IV) (*unlabeled NPs (reference)*), after labeling using $SnCl_2$ as reducing agent which was carried out at room temperature (*^{99m}Tc(IV)-NPs after labeling, $SnCl_2$, RT*), after labeling using $SnCl_2$ as reducing agent, which was carried out at 37 °C, before (*^{99m}Tc(IV)-NPs after labeling, $SnCl_2$, 37 °C*) and after purification using a PD-10 column (*^{99m}Tc(IV)-NPs after PD-10 column purification, $SnCl_2$, 37 °C*), after labeling using $Na_2S_2O_4$ as reducing agent before (*^{99m}Tc(IV)-NPs after labeling, $Na_2S_2O_4$*) and after purification using a PD-10 column (*^{99m}Tc(IV)-NPs after PD-10 column purification, $Na_2S_2O_4$*).

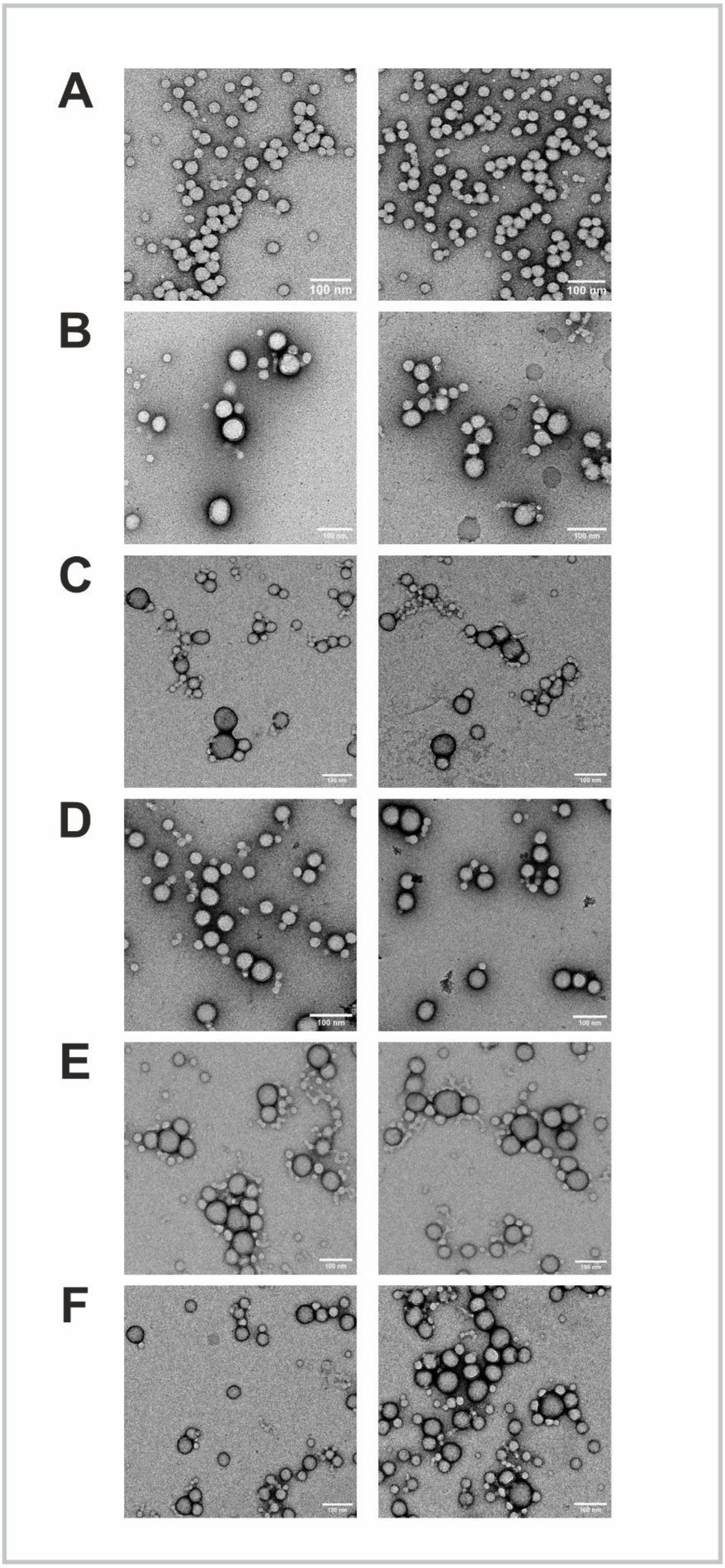

Figure S11. TEM images (A) Unlabeled (as reference), (B) ^{99m}Tc-NPs, [1] $SnCl_2$, RT, 30 min, (C) ^{99m}Tc-NPs, [2] $SnCl_2$, 37 °C, 30 min, after labeling, (D) ^{99m}Tc-NPs, [2] $SnCl_2$, 37 °C, 30 min, after PD-10 column purification, (E) ^{99m}Tc-NPs, [3] $Na_2S_2O_4$, 37 °C, 30 min, after labeling, (F) ^{99m}Tc-NPs, [3] $Na_2S_2O_4$, 37 °C, 30 min, after PD-10 purification.

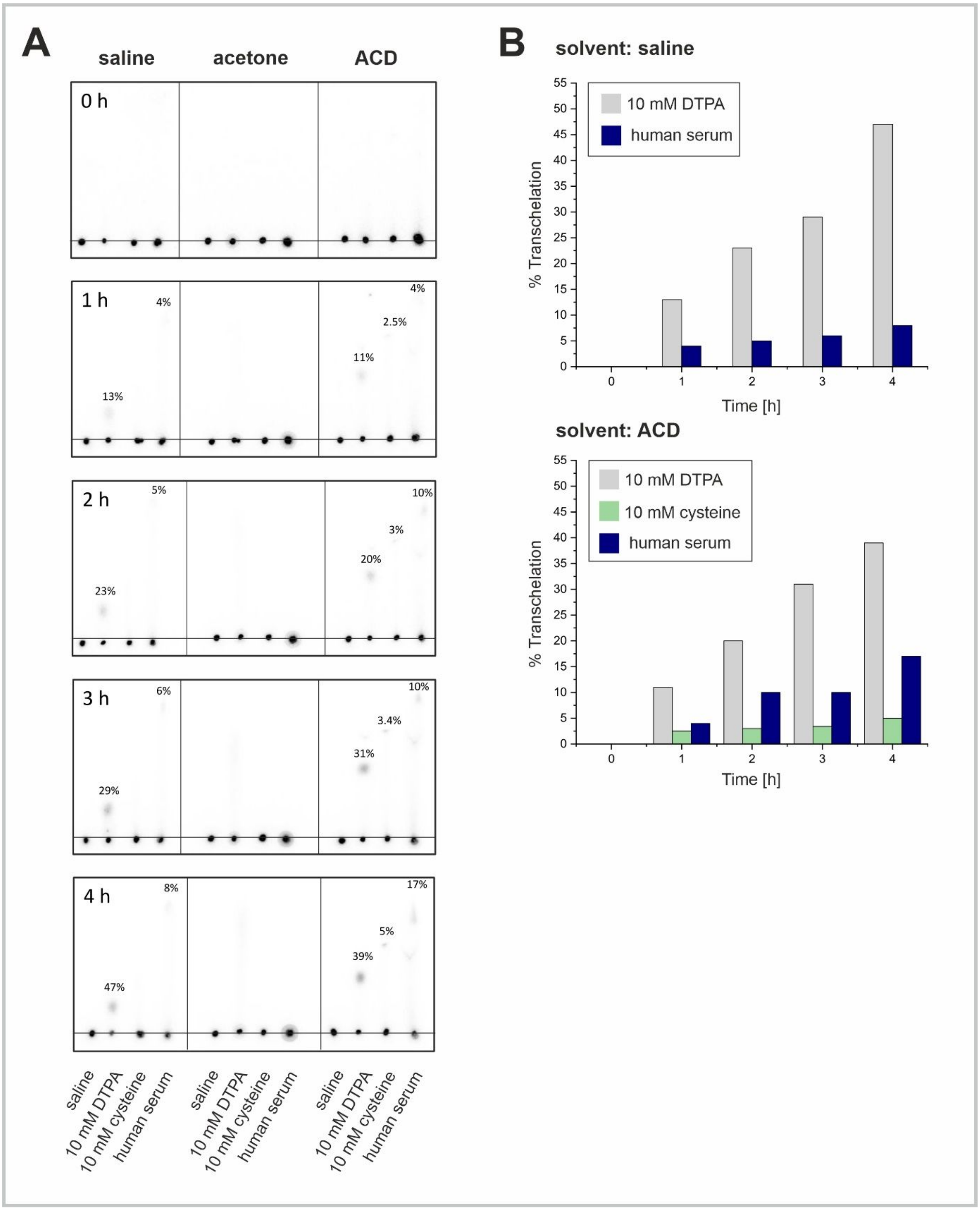

Figure S12. Results of in vitro stability and challenge tests. In vitro stability and challenge tests with saline, 10 mM DTPA, 10 mM cysteine, and human serum were performed, revealing that ^{99m}Tc-NPs were relatively stable in human serum, with the majority radioactivity remain bound to the NPs after 4 hours of co-incubation. According to published physiologically based pharmacokinetic (PBPK) models, comparable polymer NPs circulate in the bloodstream for several hours after intravenous administration and accumulate particularly in the organs of the reticuloendothelial system[18]. (A) TLCs of ^{99m}Tc-NPs mixed with 1) saline, 2) 10 mM DTPA, 3) 10 mM cysteine, 4) human serum, incubated at 37 °C for 1-4 h. Saline, acetone and ACD were used as developing solvents. ^{99m}Tc transchelated in DTPA, cysteine or serum components is eluted with aqueous solvents (saline and ACD), but not with organic solvents (acetone). (B) Percentage representation of transchelation.

Abbreviations

ACD solution	Solution of 0.068 M citrate, 0.074 M dextrose, adjusted to pH 5
$CDCl_3$	Deuterated chloroform
CY-5	Cyanine-5-amine
DBU	1,8-diazabicyclo[5.4.0]undec-7-ene
DCM	Dichloromethane
DIPEA	*N,N*-diisopropylethylamine
DLS	Dynamic light scattering
DMF	*N,N*-dimethylformamide
DPBS	Dulbecco´s phosphate-buffered saline
FTIR	Fourier-transformed infrared spectroscopy
HBTU	(2-(1H-benzotriazol-1-yl)-1,1,3,3-tetramethyluronium hexafluorophosphate
HCl	Hydrochloric acid
^{1}H-NMR	Hydrogen-1 nuclear magnetic resonance spectroscopy
HR-^{99m}Tc	Hydrolyzed-reduced technetium-99m
%ID/g	Percentage of injected dose per gram of tissue
Milli-Q water	Ultrapure water
^{99}Mo/^{99m}Tc generators	Molybdenum-99/ technetium-99m generator
mPEG5k	Poly(ethylene glycol) monomethyl ether with an average molecular weight of 5000 g mol^{-1}
$Na_2S_2O_4$	Sodium dithionite
$NaHCO_3$	Sodium bicarbonate
NPs	Nanoparticles
PDI	Polydispersity index
PEG	Polyethylene glycol
PLA-PEG	Poly(ethylene glycol)-b-poly(D, L-lactide)
PLGA	Poly(D,L)-lactide-co-glycolide
Py:AA:H_2O	Pyridine/ acetic acid/ water (3:5:1.5, v/v)
RT	Retention time
$SnCl_2$	Tin chloride
SPECT/CT	Single-photon emission computed tomography/ computer tomography
SPECT	Single-photon emission computed tomography
TEM	Transmission electron microscopy
TLC	Thin layer chromatography
^{99m}Tc-NPs	Technetium-labeled polymer nanoparticles
$^{99m}TcO_4^-$	Pertechnetate

References

(1) Spies, H., and Pietzsch, H.-J. (2007) Stannous Chloride in the Preparation of 99mTc Pharmaceuticals. in *Technetium-99m pharmaceuticals. Preparation and quality control in nuclear medicine ; with 29 tables.* (Zolle, I., Ed.), Springer, Berlin.

(2) Banerjee, T., Singh, A. K., Sharma, R. K., and Maitra, A. N. (2005) Labeling efficiency and biodistribution of Technetium-99m labeled nanoparticles: interference by colloidal tin oxide particles, *International Journal of Pharmaceutics 289,* 189–195 published online Dec 25, 2004. DOI: 10.1016/j.ijpharm.2004.09.022.

(3) Wiberg, E., and Wiberg, N. (2017) Anorganische Chemie. 103rd ed., de Gruyter, Berlin, Boston.

(4) Yi, X., Xu, M., Zhou, H., Xiong, S., Qian, R., Chai, Z., Zhao, L., and Yang, K. (2018) Ultrasmall Hyperbranched Semiconducting Polymer Nanoparticles with Different Radioisotopes Labeling for

Cancer Theranostics, *ACS nano 12,* 9142–9151 published online Sep 7, 2018. DOI: 10.1021/acsnano.8b03514.

(5) Yi, X., Shen, M., Liu, X., Gu, J., Jiang, Z., Xu, L., and Yang, K. (2021) Diagnostic Radionuclides Labeled on Biomimetic Nanoparticles for Enhanced Follow-Up Photothermal Therapy of Cancer, *Advanced healthcare materials 10,* e2100860 published online Jul 14, 2021. DOI: 10.1002/adhm.202100860.

(6) Yadav, A. K., Mishra, P., Jain, S., Mishra, P., Mishra, A. K., and Agrawal, G. P. (2008) Preparation and characterization of HA–PEG–PCL intelligent core–corona nanoparticles for delivery of doxorubicin, *Journal of Drug Targeting 16,* 464–478. DOI: 10.1080/10611860802095494.

(7) Oumzil, K., Khiati, S., Camplo, M., Koquely, M., Chuttani, K., Chaturvedi, S., Mishra, A. K., and Barthélémy, P. (2014) Nucleolipids as building blocks for the synthesis of 99m Tc-labeled nanoparticles functionalized with folic acid, *New J. Chem. 38,* 5240–5246. DOI: 10.1039/C4NJ00559G.

(8) Snehalatha, M., Venugopal, K., Saha, R. N., Babbar, A. K., and Sharma, R. K. (2008) Etoposide loaded PLGA and PCL nanoparticles II: biodistribution and pharmacokinetics after radiolabeling with Tc-99m, *Drug delivery 15,* 277–287. DOI: 10.1080/10717540802006500.

(9) Abstiens, K., Gregoritza, M., and Goepferich, A. M. (2019) Ligand Density and Linker Length are Critical Factors for Multivalent Nanoparticle-Receptor Interactions, *ACS applied materials & interfaces 11,* 1311–1320 published online Dec 20, 2018. DOI: 10.1021/acsami.8b18843.

(10) Qian, H., Wohl, A. R., Crow, J. T., Macosko, C. W., and Hoye, T. R. (2011) A Strategy for Control of "Random" Copolymerization of Lactide and Glycolide: Application to Synthesis of PEG-b-PLGA Block Polymers Having Narrow Dispersity, *Macromolecules 44,* 7132–7140. DOI: 10.1021/ma201169z.

(11) Zhao, H., Liu, Z., Park, S.-H., Kim, S.-H., Kim, J.-H., and Piao, L. (2012) Preparation and Characterization of PEG/PLA Multiblock and Triblock Copolymer, *Bulletin of the Korean Chemical Society 33,* 1638–1642. DOI: 10.5012/bkcs.2012.33.5.1638.

(12) Zimmer, O., and Goepferich, A. (2023) How clathrin-coated pits control nanoparticle avidity for cells, *Nanoscale horizons 8,* 256–269 published online Jan 30, 2023. DOI: 10.1039/d2nh00543c.

(13) Childs, C. E. (1975) The determination of polyethylene glycol in gamma globulin solutions, *Microchemical Journal 20,* 190–192. DOI: 10.1016/0026-265X(75)90038-7.

(14) Schindelin, J., Arganda-Carreras, I., Frise, E., Kaynig, V., Longair, M., Pietzsch, T., Preibisch, S., Rueden, C., Saalfeld, S., Schmid, B., Tinevez, J.-Y., White, D. J., Hartenstein, V., Eliceiri, K., Tomancak, P., and Cardona, A. (2012) Fiji: an open-source platform for biological-image analysis, *Nature methods 9,* 676–682 published online Jun 28, 2012. DOI: 10.1038/nmeth.2019.

(15) Bordes, C., Fréville, V., Ruffin, E., Marote, P., Gauvrit, J. Y., Briançon, S., and Lantéri, P. (2010) Determination of poly(ε-caprolactone) solubility parameters: Application to solvent substitution in a microencapsulation process, *International Journal of Pharmaceutics 383,* 236–243. DOI: 10.1016/j.ijpharm.2009.09.023.

(16) Maslanka Figueroa, S., Fleischmann, D., Beck, S., and Goepferich, A. (2020) Thermodynamic, Spatial and Methodological Considerations for the Manufacturing of Therapeutic Polymer Nanoparticles, *Pharmaceutical research 37,* 59 published online Feb 24, 2020. DOI: 10.1007/s11095-020-2783-4.

(17) Tang, S., Zhang, R., Liu, F., and Liu, X. (2015) Hansen solubility parameters of polyglycolic acid and interaction parameters between polyglycolic acid and solvents, *European Polymer Journal 72,* 83–88. DOI: 10.1016/j.eurpolymj.2015.09.009.

(18) Li, M., Panagi, Z., Avgoustakis, K., and Reineke, J. (2012) Physiologically based pharmacokinetic modeling of PLGA nanoparticles with varied mPEG content, *International journal of nanomedicine 7,* 1345–1356 published online Mar 7, 2012. DOI: 10.2147/IJN.S23758.
